# Developing a model to predict neonatal respiratory distress syndrome and affecting factors using data mining: A cross-sectional study

**DOI:** 10.18502/ijrm.v21i11.14654

**Published:** 2023-12-19

**Authors:** Parisa Farshid, Kayvan Mirnia, Peyman Rezaei-Hachesu, Elham Maserat, Taha Samad-Soltani

**Affiliations:** ^1^Department of Health Information Technology, School of Management and Medical Informatics, Tabriz University of Medical Sciences, Tabriz, Iran.; ^2^Department of Pediatrics, Children's Medical Center, Tehran University of Medical Sciences, Tehran, Iran.; ^3^Department of Medical Informatics, School of Medical Sciences, Tarbiat Modares University, Tehran, Iran.

**Keywords:** Data mining, Classification, Neonatal respiratory distress syndrome, Newborn, Machine learning.

## Abstract

**Background:** One of the major challenges that hospitals and clinicians face is the early identification of newborns at risk for adverse events. One of them is neonatal respiratory distress syndrome (RDS). RDS is the widest spared respiratory disorder in immature newborns and the main source of death among them. Machine learning has been broadly accepted and used in various scopes to analyze medical information and is very useful in the early detection of RDS. **Objective:** This study aimed to develop a model to predict neonatal RDS and affecting factors using data mining. **Materials and Methods:** The original dataset in this cross-sectional study was extracted from the medical records of newborns diagnosed with RDS from July 2017-July 2018 in Alzahra hospital, Tabriz, Iran. This data includes information about 1469 neonates, and their mothers information. The data were preprocessed and applied to expand the classification model using machine learning techniques such as support vector machine, Naïve Bayes, classification tree, random forest, CN2 rule induction, and neural network, for prediction of RDS episodes. The study compares models according to their accuracy. **Results:** Among the obtained results, an accuracy of 0.815, sensitivity of 0.802, specificity of 0.812, and area under the curve of 0.843 was the best output using random forest. **Conclusion:** The findings of our study proved that new approaches, such as data mining, may support medical decisions, improving diagnosis in neonatal RDS. The feasibility of using a random forest in neonatal RDS prediction would offer the possibility to decrease postpartum complications of neonatal care.

## 1. Introduction

Early identification of infants at risk of adverse conditions is one of the biggest challenges that hospitals and clinicians face. One of them is neonatal respiratory distress syndrome (RDS). RDS accounts for a significant proportion of neonatal mortality and morbidity, and usually occurs in the days after birth. In RDS, effective prevention and treatment strategies are available with early detection of the disease contributing to better prognosis (1).On the other hand, unnecessary treatment of newborns who may show the first signs of illness, such as the adverse effects of ventilators when RDS is suspected, can harm the patient. Therefore, physicians still require new and improved methods to quickly and accurately detect infants at risk of undesirable outcomes (2).

Data mining (DM) techniques and machine learning (ML) algorithms play a very important role in medicine. DM applications are to make better health policies and prevention of hospital errors, early detection and prevention of diseases, and reduction in hospital mortality rates (3).

Several studies in the neonatal field have used supervised learning methods, for example, support vector machine (SVM), artificial neural network, decision tree, K-nearest neighbor (KNN), and random forests have been used in diagnosing and predicting neonatal diseases, such as jaundice (4-8), extubation failure for neonates with RDS (9-11), neonatal death (12), RDS and hypoglycemia, infant mortality (13), low birth weight (14-18), apnea (19), neonatal resuscitation, early postoperative survival in infant heart transplantation (20), metabolic disorder and prematurity (21).

As neonatal medicinal service suppliers need to get to guidelines and a clinical decision support system; therefore, there is a considerable preference to utilize and adjust the present-day advancements (22).

A research demonstrated the suitability of DM models (DMM) to forecast neonatal death in neonatal intensive care units (12). The boosted trees and logistic regression models were used to predict neonatal RDS and hypoglycemia before discharge (23).

The application of DM techniques can be an effective way to improve the prediction of newborns diseases. Besides, it embosses supervised learning techniques for neonatal data investigation with various ways to increase model accuracy. Information about the risk factors of RDS enables healthcare professionals to identify high-risk neonates. An accurate evaluation of the risk factors can result in the prediction of the essential resources and staff to accomplish newborns resuscitation. The rapid and effective resuscitation can be crucial for neonatal health, particularly for the prevention of hypoxic organ harm or even brain harm.

Therefore, providing a model/decision support system based on DM techniques can be beneficial in relieving risk factors and improving infants' health conditions by using its capability in exact, accurate, real-time, and rapid anticipation of the RDS. Additionally, it could accomplish all the needed procedures right after the infant's birth, fulfilling the accumulated care's performance and decrease medical errors. According to our information, no other study used DM techniques to predict RDS and its risk factors in the neonatal population. Hence, this study aims to predict neonatal RDS and affecting factors with applying DM.

## 2. Materials and Methods

### Data collection and selection

The original dataset in this cross-sectional study was extracted from the medical record of newborns diagnosed with RDS between July 2017 and July 2018 in Alzahra hospital of Tabriz, Iran. This data includes information about 1469 newborns and their mothers. The data collecting tool in this research was our searcher-made checklist approved by the neonatal associate professor, and the data was transcribed into a Microsoft Excel database, which was prepared earlier for this objective. In total, 20 variables were gathered and analyzed.

The methodology of the current study conformed to the different phases of the cross-industry standard process (CRISP-DM) for DM model (24). For this study, all algorithms were applied using Orange, an open-source DM and visualization software with strenuous association. It provides the design of the data analysis process via user-friendly visual programming.

### Business understanding

The business goals of the current research were the prediction of RDS in neonatal, considering the infants' specification and likewise given the sort of delivery, the specification of the pregnancy, and the well-being states of the mother. This prediction must be delicate and precise since it can be critical for the infant's life. Furthermore, anticipating in advance that an infant will require enhanced consideration can enable obstetricians to deal with their time and endeavors better and, in this manner, convey increasingly viable considerations to babies.

### Data comprehension

The data file explanation was applied to increase comprehension of the features. The target variable RDS represents whether the newborn has RDS and binary values: yes or no.

Initially, the dataset contained 20 predictor features and 1469 rows. The quantitative features consist of the mother age, birth weight, Apgar score at $1^{\text{st}}$ and $5^{\text{th}}$ min, newborn head circumference, and length. Maternal covariates include mode of delivery, blood group, hypertension, preeclampsia, diabetes, thyroid, neonatal steroid, premature rupture of the membranes, and magnesium sulfide. The infants variables include gender, blood group, meconium aspiration syndrome, premature, and RDS being the target variable.

#### Data exploration and preprocessing

Clinical data are rarely in a structured and clean form that can be used for many ML algorithms. This period of the DM procedure included the election and provision of the data to be improvised to the DMM.

In this study, data preprocessing involved the following steps: 1) missing data management, 2) discretization, and 3) dimensionality reduction or feature selection. In all phases, hyper parameters selection was performed in several trials until reaching optimal outcomes. Missing data were imputed by average/most frequent. The information gain, Gini index, and gain ratio methodologies were used for the dimensionality reduction, and the top 7 attributes were chosen. The entropy discretization strategy was used for continuous variables in this study. For further understanding the significance of the input features, it is common to analyze the effect of input features during neonatal RDS prediction, in which the effect of specific input features of the model on the output features has been analyzed. Tests were directed applying 3 tests to evaluate input features: information gain test, gain ratio test, and Gini index test. Various algorithms obtain very different results, that is, each of them describes the relation of variables differently. The average value of all the algorithms is taken as the last outcome of features ranking, rather than choosing one algorithm based on it. The results acquired with these values are offered in table I.

The following analysis is to specify the value of each variable exclusively. The variables which were highly influenced are prematurity, birth weight, prenatal steroid and head circumference, and length; Apgar 1 and Apgar 5 were weakly influenced predictors.

Table I indicates that feature prematurity showed the best efficiency in all 3 tests. Also, the gain ratio test enhances the accuracy of the prediction of the models; therefore, by selecting 7 top features, it was purposed as the superior test for rating features.

### Modeling

After the preprocessing was completed, the next step was to apply a sample of the dataset for training and testing algorithms. Using k = 10, the cross-validation method was applied. Samples were divided into k equal subsets for this procedure. The model was then trained k times, rejecting one fold per cycle. 9 folds were applied to each circle for training purposes, and the remaining folds were used to test DM algorithms.

The second step after preprocessing, followed by the application of a sample of the dataset for training and testing algorithms. Using k = 10, the cross-validation method was applied. Samples were divided into k equal groups for this approach. The model was then trained k times, rejecting one fold per cycle. 9 folds were applied to each circle for training purposes, and the remaining folds were utilized to test DM algorithms.

2 sampling techniques were analyzed for each DM method: cross-validation using 10 folds, where all data is used for testing, and random sampling, where 70% of the data is used for training and the remaining sum for testing. Moreover, 2 data strategies were tested: with or without oversampling and with or without feature selection of all cases. There was just a single target variable, which was the *RDS *variable, and the contemplated scenarios were the following:

1. Random sampling with oversampling and feature selection

2. Random sampling with oversampling and without feature selection

3. Random sampling without oversampling and feature selection

4. Random sampling without oversampling and with feature selection

5. Cross-validation with oversampling and feature selection

6. Cross-validation with oversampling and without feature selection

7. Cross-validation without oversampling and feature selection

8. Cross-validation without oversampling and with feature selection

Different `DM' algorithms have been applied to predicting neonatal RDS, including KNN, Naive Bayes, random forest, SVM, neural network, classification tree, and CN2 rule induction.

### Evaluation

The execution of each DMM was evaluated via its confusion matrix, which offers the number of true positives (TP), false positives (FP), true negatives (TN), and false negatives (FN). With these outcomes, it is conceivable to compute sensitivity, specificity, and accuracy to evaluate the algorithm's performance.

Accuracy refers to the percentage of correctly classified records: 


$$\text{Accuracy}=\frac{\text{TP}+\text{TN}}{\text{Positive(P)}+\text{Negative(N)}}$$

Sensitivity is otherwise called the true positive rate or recall; this is the proportion of the number of positive instances arranged totally as the positive instances (25). 


$$\text{Sensitivity}=\frac{\text{TP}}{\text{TP}+\text{FN}}$$

Specificity is used for the goal of measuring the extent of negative cases that were accurately arranged as negative, which is 1-FP (false positive), (25, 26) or can be determined as follows: 


$$\text{Specificity}=\frac{\text{TN}}{\text{TN}+\text{FP}}$$

The receiver operating characteristic (ROC) curve is a 2-dimensional diagram demonstrating the ratio of false positive and true positive rates. On a ROC curve, the X-axis shows the percent of the FP (1-specificity) = FP/ (TN + FP), and the Y-axis shows the TP (sensitivity) = TP/ (TP + FN). The AUC is a standard efficiency measured for a ROC curve. It obtains any amount between (0, 1).

To implement algorithms in clinical practice, we developed a web-based user interface (UI) on top of the DM platform. Fortunately, Orange software is a free and open-source platform programed by Python. It allowed us to use Python codes instead of graphical widgets to develop our UI and customized software. The documentation of the Orange developer was used to reach this aim. Finally, a simple web view UI was designed to access practitioners to the system across smart phones.

**Table 1:** Results of dimensionality reduction by using 3 prevalent methods and selecting the top 7 candidate features


**Attributes**							
**Criteria**	**Rank1**	**Rank2**	**Rank3**	**Rank4**	**Rank5**	**Rank6**	**Rank7**
**Information gain**	Prematurity 0.326	Birth weight 0.076	Prenatal steroid 0.061	HC 0.048	Length 0.033	Apgar 1 0.028	Apgar 5 0.009
**Gain ratio**	Prematurity 0.463	Birth weight 0.089	Prenatal steroid 0.066	HC 0.036	Length 0.047	Apgar 1 0.040	Apgar 5 0.025
**Gini index**	Prematurity 0.183	Birth weight 0.044	Prenatal steroid 0.037	HC 0.028	Length 0.020	Apgar 1 0.017	Apgar 5 0.006
HC: Head circumference							

### Ethical considerations

This study was approved by the Ethical Committee of Tabriz University of Medical Sciences, Tabriz, Iran (Code: IR.TBZMED.REC.1399.692).

## 3. Results

**Figure 1:**
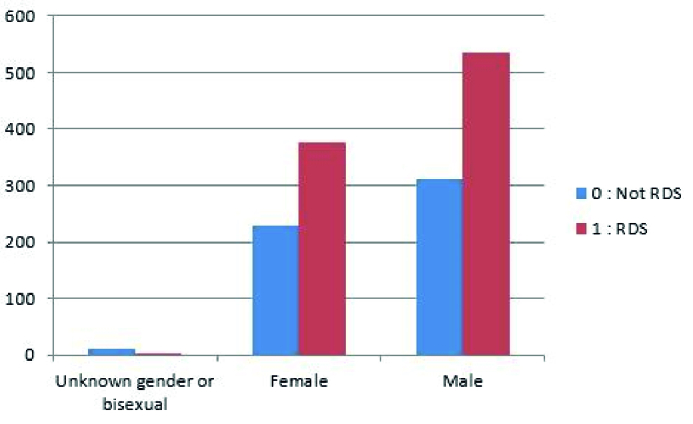
Gender distribution of the RDS variable. RDS: Respiratory distress syndrome.

Neonatal resuscitation was required for 90% of the registered newborns, as shown from figure 1, which displays the data distribution of the RDS variable on the used dataset. Figure 1 indicates the data dissemination of gender variables on the utilized dataset, and likewise, it might be observed, 53.4% of the registered male newborns needed neonatal resuscitation.

RDS frequency distributions were 87.79% in premature infants, 61.75% in infants under the weight of 1525 gr, and 57.23% in infants who did not receive prenatal steroids.

Various DM algorithms, including tree, SVM, neural network, random forest, KNN, Naive Bayes, and CN2 rule induction, were applied to the prediction of RDS. Predictive model development is a repetitive process, and, as a result, it is decisive to accomplish several experiences with various classifiers to choose the best model for solving the problem at hand. 10-fold cross-validation was used to obtain model accuracy and area under the curve (AUC). The models' efficiency was evaluated and compared using various metrics, including accuracy, sensitivity, specificity, and AUC. The algorithm's overall performance is expressed as an indicator by the AUC (27). The models that had the highest AUC were therefore considered to be the best (28). Of all the instances, 70% was used for training, and the remaining 30% constructed the test set. The accuracy measures of different DM algorithms with different validation techniques are depicted in table II.

For the prediction of neonatal RDS, it has been noted that cross-validation method test without over sampling and with feature selection on train data, the random forest, neural network, and classification tree, results in a higher overall prediction accuracy, specificity, sensitivity, and AUC comparison with other classification methods. Also, SVM gives a lower overall prediction accuracy (72.7%). The CN2 rule induction had lower sensitivity (63.2%) than the other algorithms. Random forest had the highest specificity (81.2).

**Figure 2:**
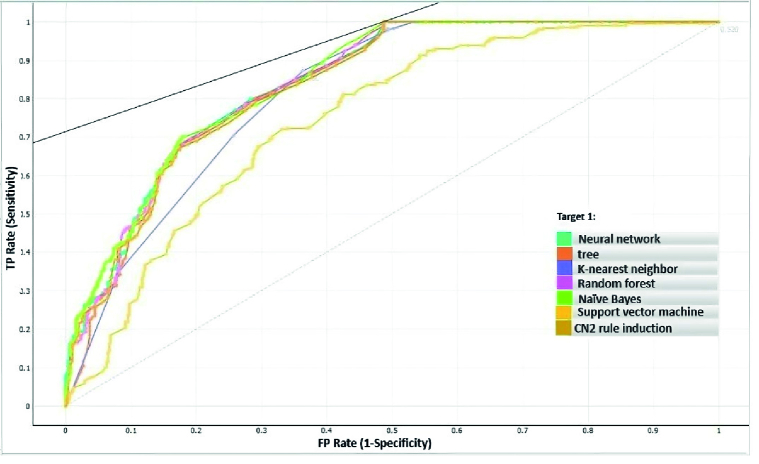
ROC curve for the current study's machine learning algorithms; the colors represent various learning strategies. ROC: Receiver operating characteristic, TP: True positives, FP: False positives.

This means that random forest has the highest score in the correct prediction of non-RDS infants. According to the results, the highest sensitivity is also related to random forest (80.2%). Sensitivity is a great necessity in correctly diagnosing the disease (15). As shown in figure 2, random forest algorithm had the highest performance (84.3%) approach of AUC in predicting neonatal RDS.

There are a number of decision tree structures in the Random Forest classification. In order to sample the trees that are associated with this classifier, it uses a random scheme (29). It is one of the most widely used analytical tools with high prediction accuracy. This algorithm is superior to several other classical algorithms because of its ease of implementation, efficiency when working with complex datasets, and ability to handle datasets with varying sample sizes (30).

**Figure 3:**
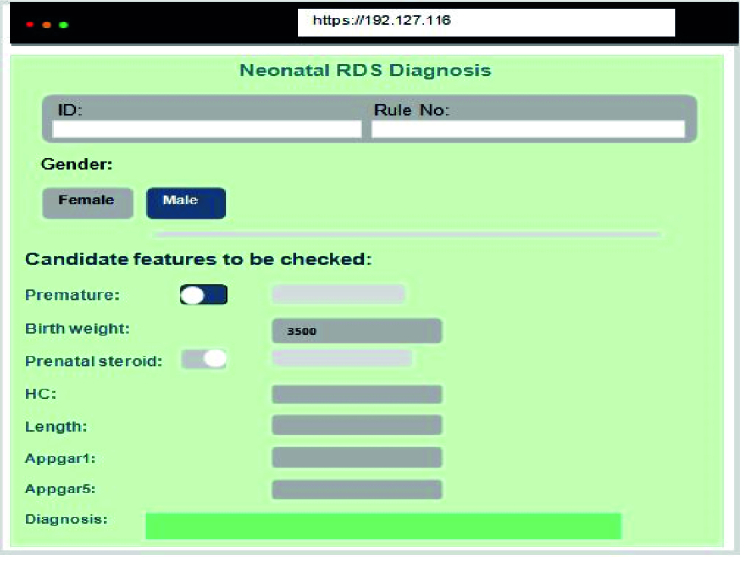
User interface. RDS: Respiratory distress syndrome, HC: Head circumference.

The UI of the developed system is displayed in figure 3. It was coded using PHP and Orange libraries in Python 3.7. To exchange data between these different platforms, we used JSON web services. The system recommends a differential diagnosis of neonatal RDS.

**Table 2:** Predictive performance of various classification methods


**Algorithm**	**Accuracy**	**Sensitivity**	**Specificity**	**AUC**
**Tree**	0.812	0.714	0.810	0.835
**SVM **	0.727	0.684	0.736	0.746
**Neural network**	0.813	0.741	0.811	0.843
**Random forest**	0.815	0.802	0.812	0.843
**KNN**	0.784	0.760	0.790	0.817
**Naive bays**	0.760	0.715	0.781	0.847
**CN2 rule induction **	0.809	0.632	0.807	0.836
AUC: Area under the curve, SVM: Support vector machine, KNN: K-nearest neighbor, CN2: Clark and Niblett 1989				

## 4. Discussion

Using cross-validation as the sampling method produced better results than random sampling, according to the analysis of the collected data. The algorithms that had the top accuracy consequences, respectively, were random forest (81.5%), neural network (81.3%), and classification tree (81.2%).

The model that employed the cross-validation sampling method, the classification tree, the 8 scenario, feature selection, and no oversampling of the data was deemed to be the most suitable, as it had the highest sensitivity value. This study's results indicate that the most important factor in predicting RDS was prematurity.

Comparing with the literature, most of the antecedent research had concentrated on predicting low birth weight and its risk factors (15-17). Only one study has examined the prediction of RDS before discharge (23). Different data classification algorithms was compared to determine the type of jaundice in neonates (31). The results of the studies are compared in table III.

The present paper was conducted with the study of Safdari et al., in terms of the software (Orange) used for DM (31). In this study, the algorithm with the highest accuracy of prediction was the random forest (0.815) which was similar other studies (accuracy = 0.980, 0.880) (17, 19). In our study, the variables were 21 (with RDS as the target variable).

In the various studies, the number of variables has been reported between 8-528 (8, 12-20, 23, 31-34). The study showed that the number of variables had no relationship with the accuracy of algorithm prediction. Several studies have used the highest number of samples in their datasets (i.e., 154755, 10000, 7800, 4498, 3163, 2386, 1762, and 1348) (11-13, 16, 18, 20, 23, 34).

This is while our study was on the 8 platform (1469 samples). In a study with 261 samples had the highest prediction accuracy (98.60%) (17). Therefore, the higher sample size did not affect the accuracy of algorithm prediction. In this paper, the sensitivity range was between 0.632 and 0.802 in various algorithms, while the highest sensitivity was for random forest (0.815). According to Senthilkumar and Paulraj's studies the highest sensitivity belongs to random forest (0.9923) (15). This means that the random forest predicts TP cases with a higher percentage, which is very important in terms of diagnostic value. In our study, the highest specificity was reached by the random forest algorithm (0.812), while the specificity of KNN was more than 0.994 in other study (20).

According to the information from other studies presented in table III, the highest rates of specificity with different algorithms were 0.994, 0.980, 0.970, and 0.9923, respectively, which indicated that the type of algorithm could not be involved in increasing the prediction of TN. Also, the entire information of table III, proved that the higher specificity in an algorithm with low sensitivity were not valuable.

**Table 3:** Comparison of current work with literature


			**Utility (%)**			
**Author, year (Ref)**	**Data set**	**Target**	** Features used**
				for prediction	**Software**	**Classifiers**	**Accuracy**	**Sens**	**Spec**	**AUC**
			Random forest	81.5			80.2	81.2	84.3
			Neural network	81.3	74.1	81.1	84.3
			Decision tree (C4.5)	81.2	71.4	81	83.5
			CN2 rule induction	80.9	63.2	80.7	83.6
**Farshid ** ***et al.*** **,**
	**(present study)**	1469 neonates
	admitted to	
	NICU		RDS	Demographic,
	maternal,			
physiological				
parameters (with				
21 features)		KNN			78.4	76	79	81.7
				Logistic regression	NR	NR	NR	81.37
**Natarajan ** ***et al.*** **,**	
		**2023 (11)**	1348 low birth
weight			
neonates	Extubation
failure for	
low-birth-weight
neonates	Demographics,
vital signs,	
ventilator
parameters, and
medication	NR	Booted tree	NR	NR	NR	82.27
				Neural network	86	86	83	92
			Decision tree (C4.5)	84	82	89	91
			SVM	82	82	78	89
**Sheikhtaheri ** ***et al.*** **,**
	**2021 (12)**	1762 records
	collected from	
	a neonatal	
registry		
database	Deaths	Neonatal and
maternal data		
(with 17 features)			IBM SPSS
Modeler				Random forest	63.5	61	72	81
				Logistic regression	NR	72.5	NR	78.8
				Naive Bayes	NR	72.5	NR	78.5
**Hajipour ** ***et al.*** **,**
	**2021 (13)**	Health records
	of 2386	
mothers	Mortality		Neonatal and
maternal data			
(with 16 features)		STATA-version 14	
and Python 2.7.		
IDLE software	Random forest		NR	72.3	NR	77.7
			Boosted trees	NR	NR	NR	92.3
**Betts ** ***et al.*** **,**
	**2021 (23)**	Medical record
of 154,755		
neonates	RDS and
hypoglycemia		Neonatal and
maternal data		
(with 528 features)	R software	Logistic regression	NR	NR	NR	89.9
				Neural network (MLP)	68.16	81.8	NR	NR
			SVM	80.29	80.3	NR	NR
**Borson ** ***et al.*** **,**
	**2020 (18)**	4498 instances
	collected from	
the website		
of “the DHS
program"	Low birth weight	Neonatal and
maternal DHS		
data (with 10		
features)	NR	Logistic	80.30	80.3	NR	NR
**Daunhower ** ***et al.*** **, 2019 (8)**		Data of 362 neonates	Hyperbilirubin-emia	Neonates` clinical data (with 20 features)	Python3	Random forest	NR	NR	NR	93.29
				Decision tree (J48)	98	98	97.8	98.3
			Simple Bayes	96.3	96	97.5	99.1
**Ghaderi-Ghahfarokhi**
	***et al.*** **, 2018 (17)**	Medical
	records of 261	
neonates	Low birth weight		Demographic,
neonatal,			
and maternal			
characteristics
(with 18 features)	Weka	Random forest	98.9	94.8	98	99.9
				The J48	NR	66.3	95.4	90.3
				REP Tree	NR	61.7	94.9	88.4
**Hange ** ***et al.*** **,**
	**2017 (16)**	10,000 births
	records		Birth weight group	Demographic,
maternal,				
neonatal, and				
other condition
(with 90 features)	Weka	Random tree	NR	65	95.5	85.3
**Morais ** ***et al.*** **, 2017 (20)**		3163 newborns` information from EHR	Need for neonatal resuscitation	Baby's characteristics and the characteristics of the pregnancy and the health conditions of the mother (with12 features)	Weka	KNN, cross-validation, with oversampling	98.48	90	99.4	NR
for prediction		**Software**	**Classifiers**	**Accuracy**	**Sens**	**Spec**	**AUC**
**Chen ** ***et al.*** **, 2018 (32)**	48 infants listed for heart transplantation			Post-operative survival in infant heart transplantation	Infants` demographics, clinical characteristics at the listing, and outcomes (with 17 features)	CART analysis software	CART	83	95	76	83
**Safdari ** ***et al.*** **, 2018 (31)**	325 neonates with jaundice admitted to NICU	Indirect jaundice, jaundice indirect then turn into direct jaundice, and direct jaundice	Demographic and neonatal characteristics (with 17 features)	Orange	Classification tree	94.2	94.2	NR	87.3
			Random forest	88	NR	NR	NR
			Boosted C5.0 model	78	NR	NR	NR
			Decision tree (C5.0)	69	65	70	NR
**Shirwaikar ** ***et al.*** **,**
	**2016 (19)**	229 neonates
	admitted to	
	NICU		Neonatal apnea	Demographic,
maternal,				
and neonates'				
physiological				
parameters (with
23 features)	R software	SVM	77	61	97	NR
				Classification tree	89.95	97.69	72.88	93.80
				Logistic regression	74.07	92.31	33.90	77.24
				Naive Bayes	77.78	90	50.85	80.08
			Random forest	70.90	99.23	80.47	84.20
			Neural network	74.19	93.85	37.29	78.04
**Senthilkumar **
	***et al.*** **, 2015 (15)**	The collected
	data set	
	of 189 women		Low-birth weight	Maternal
clinical				
information				
(with 11				
features)	NR	SVM			74.07	98.46	20.34	77.38
**Najafian ** ***et al.*** **, 2015 (14)**		45 neonates with RDS			INSURE failure in low birth weight	Infants` demographic, para-clinical, and clinical information (with 15 features)	SPSS	Logistic regression	NR	NR	NR	NR
				Simple logistic naive	NR	90	56	89
				Naive Bayes	NR	90	56	88
**Ferreira ** ***et al.*** **,**	
		**2012 (33)**	227 infants	Neonatal jaundice	Mother, father,
	siblings				
information,					
and infant
clinical
information
(with 72
features)	Weka	Bayes net.	NR	90	60	87
**Mikhno & Ennett 2012 (34)**		Over 7800 neonates	Extubation failure for neonates with RDS	Patient demographic data, neonates monitoring, chart data, laboratory results, ventilator settings and values, ICD-9 codes, LOINC codes, and free text nursing progress notes (with 57 features)	NR	Logistic regression	NR	61.4	NR	83.9
Senc: Sensitivity, Spec: Specificity, AUC: Area under the curve, NICU: Neonatal intensive care unit, RDS: Respiratory distress syndrome, KNN: K-nearest neighbor, NR: Not reported, IBM SPSS: International business machines statistical package for the social sciences, SVM: Support vector machine, STATA: Statistics and data, IDLE: Integrated development and learning environment, DHS: Demographic and health surveys, MLP: Multilayer perceptron, REP: Reduced error pruning tree, EHR: Electronic health record, CART: Classification and regression tree, ICD-9: International classification of diseases $9^{\text{th}}$ revision, LOINC: Logical observation identifiers names and codes									

## 5. Conclusion

To sum up, random forest and classification trees are effective tools for neonatal RDS prediction, with key variables identified. DM proves essential but faces challenges, emphasizing the need for further research to fully harness its potential in obstetric and neonatal healthcare. Embracing new technologies like DM can support medical decisions and enhance neonatal RDS diagnosis. Future studies should explore additional features. Efforts to develop scalable predictive models from healthcare data and ML hold promise, providing valuable insights for clinical practitioners. This study has some limitations, because of the large number of samples, data collection in the hospital environment is time consuming and costly. Another limitation of this research was the busy medical and nursing staff of the neonatal intensive care unit, which resulted in collecting an unclean data set. Too much time was spent clearing and modifying the data. This problem and the inadequate familiarity of the personnel involved in the creation of the Excel database lowered the accuracy of the registration of important features and their inclusion in the database. It should be noted that various bias can arise from different stages of the DM process, such as data collection, preprocessing, analysis, and interpretation.

##  Conflict of Interest

The authors declare that there is no conflict of interest.
